# Supplementary material for: Identification of photoperiod-induced specific miRNAs in the adrenal glands of Sunite sheep (Ovis aries)
Source: Front Vet Sci. 2022 Jul 22;9:888207. doi: 10.3389/fvets.2022.888207 (PMC9354845; doi:10.3389/fvets.2022.888207)
Supplement: Supplementary file 1 [file Data_Sheet_1.ZIP › Supplementary file/miRDeep2 file.zip!/miRDeep2 file/Know_Structure/SP42A2/oar-mir-27a.pdf]

miRBase precursor : oar-mir-27a
Total read count : 404087
oar-miR-27a read count : 403676
remaining reads : 411

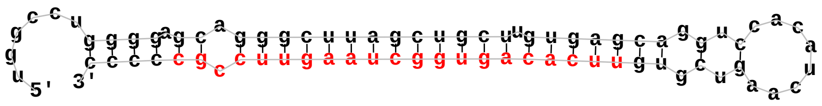

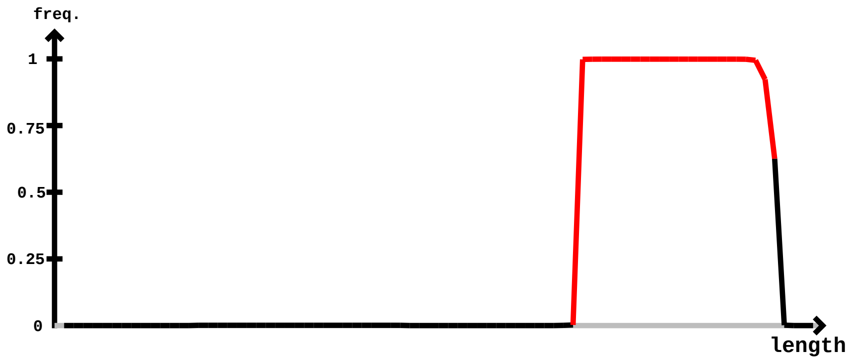

oar-miR-27a

```
5'- uggccuggggagcagggcuuagcugcuugugagcagguccacaucaagucgugUUcacaguggcuaaguuccgccccc  -3'   exp
    ......((((.((.(((((((((((((.(((((((.(.(........).).)))))))))))))))))))).))))))  reads   mm   sample
    ............cagggcuuagcugcuugugagc............................................  1       0    seq
    .............agggcuuagcugcuugug...............................................  1       0    seq
    .............agggcuuagcugcuugugag.............................................  1       0    seq
    .............agggcuuagcugcuugugagc............................................  60      0    seq
    .............agggcuuagcugcuugugagca...........................................  336     0    seq
    .............agggcuuagcugcuugugagcag..........................................  1       0    seq
    .............agggcuuagcugcuugugagcagg.........................................  8       0    seq
    .............agggcuuagcugcuugugagcaggu........................................  3       0    seq
    ...................................................uguucacaguggcuaagu.........  1       0    seq
    ...................................................uguucacaguggcuaaguu........  6       0    seq
    ...................................................uguucacaguggcuaaguuc.......  20      0    seq
    ...................................................uguucacaguggcuaaguucU......  5       1    seq
    ...................................................uguucacaguggcuaaguucc......  16      0    seq
    ...................................................uguucacaguggcuaaguuccg.....  235     0    seq
    ...................................................uguucacaguggcuaaguucUg.....  1       1    seq
    ...................................................uguucacaguggcuaaguuccgc....  33      0    seq
    ...................................................uguucacaguggcuaaguuccgcc...  1       0    seq
    ....................................................guucacaguggcuaaguuc.......  1       0    seq
    ....................................................guucacaguggcuaaguucU......  47      1    seq
    ....................................................guucacaguggcuaaguucc......  8       0    seq
    ....................................................guucacaguggcuaaguuccg.....  1       0    seq
    ....................................................guucacaguggcuaaguucUg.....  106     1    seq
    ....................................................guucacaguggcuaaguucUgc....  432     1    seq
    ....................................................guucacaguggcuaaguuccgc....  2       0    seq
    .....................................................uucacagugUcuaaguu........  1       1    seq
    .....................................................Aucacaguggcuaaguu........  3       1    seq
    .....................................................uucacaguggcuaaguG........  1       1    seq
    .....................................................uucacaguggcuaaguu........  141     0    seq
    .....................................................uucacaguggAuaaguu........  1       1    seq
    .....................................................uuGacaguggcuaaguu........  1       1    seq
    .....................................................uucacaguggUuaaguu........  1       1    seq
    .....................................................uucacaguggcuaagCu........  2       1    seq
    .....................................................uucacaguggcuaaguuc.......  1541    0    seq
    .....................................................uucacaguggcuaaguuG.......  9       1    seq
    .....................................................uucacaguAgcuaaguuc.......  1       1    seq
```

oar-miR-27a

```
uggccuggggagcagggcuuagcugcuugugagcagguccacaucaagucguguucacaguggcuaaguuccgccccc
.....................................................uucacaguggcuaaguucU......   23870    1    seq
.....................................................uucacaguggcuaaguucc......   4983     0    seq
.....................................................uucacaguggcuaaguuccA.....   16512    1    seq
.....................................................uucacaguggcuaaguucUg.....   41092    1    seq
.....................................................uucacaguggcuaaguuccg.....   62281    0    seq
.....................................................uucacaguggcuaaguuccgc....   8526     0    seq
.....................................................uucacaguggcuaaguucUgc....   242862   1    seq
.....................................................uucacaguggcuaaguuccgcc...   555      0    seq
.....................................................uucacaguggcuaaguuccgccc..   7        0    seq
......................................................ucacaguggcuaaguucc......   3        0    seq
......................................................ucacaguggcuaaguucU......   18       1    seq
......................................................ucacaguggcuaaguuccg.....   43       0    seq
......................................................ucacaguggcuaaguucUg.....   31       1    seq
......................................................ucacaguggcuaaguuccA.....   11       1    seq
......................................................ucacaguggcuaaguuccgc....   10       0    seq
......................................................ucacaguggcuaaguucUgc....   197      1    seq
.......................................................cacaguggcuaaguucU......   2        1    seq
.......................................................cCcaguggcuaaguuccg.....   1        1    seq
.......................................................cacaguggcuaaguuccg.....   10       0    seq
.......................................................cacaguggcuaaguuccA.....   2        1    seq
.......................................................cacaguggcuaaguucUg.....   5        1    seq
.......................................................cacaguggcuaaguuccgc....   1        0    seq
.......................................................cacaguggcuaaguucUgc....   25       1    seq
........................................................acaguggcuaaguucUg.....   2        1    seq
........................................................acaguggcuaaguuccg.....   2        0    seq
........................................................acaguggcuaaguucUgc....   6        1    seq
.........................................................caguggcuaaguucUgc....   2        1    seq
```
